# Biomechanics following skip-level cervical disc arthroplasty versus skip-level cervical discectomy and fusion: a finite element-based study

**DOI:** 10.1186/s12891-019-2425-3

**Published:** 2019-01-31

**Authors:** Ting-kui Wu, Yang Meng, Bei-yu Wang, Xin Rong, Ying Hong, Chen Ding, Hua Chen, Hao Liu

**Affiliations:** Department of Orthopedic Surgery, West China Hospital, Sichuan University, No. 37 Guo Xue Rd, Chengdu, 610041 Sichuan China

**Keywords:** Cervical spine, Finite element analysis, Skip-level cervical degenerative disc disease, Cervical disc arthroplasty, Intradiscal pressure

## Abstract

**Background:**

Moderately increased motion at the intermediate segment (IS) after skip-level fusion may accelerate disc degeneration. However, limited biomechanical data are available that examine the effects on the IS following cervical disc arthroplasty (CDA). The purpose of this study is to investigate the biomechanical changes in the IS of the cervical spine after skip-level fusion or skip-level arthroplasty.

**Methods:**

A finite element model of a healthy cervical spine (C2-C7) was constructed. Two surgical models were developed: (1) skip-level fusion at C3/4 and C5/6 and (2) skip-level arthroplasty at C3/4 and C5/6. A 75-N follower load and 1.0-N·m moments were applied to the top of the C2 vertebra to produce flexion, extension, lateral bending and axial rotation in the intact model. The end-points in each direction corresponding to the intact model were applied to the surgical models under displacement-control protocols.

**Results:**

The ranges of motion (ROMs) of the fusion model were markedly decreased at the operated levels, while the corresponding ROMs of the arthroplasty model were similar to those of the intact spine in all directions. In the fusion model, the ROMs of the IS (C4/5) were markedly increased in all directions. The ROMs in the arthroplasty model were similar to those in the intact spine, and the ROMs of untreated segments were evenly increased. In the fusion model, the intradiscal pressure and facet contact force at were C4/5 remarkably increased and unevenly distributed among the unfused segments. In the arthroplasty model, the IS did not experience additive stress.

**Conclusion:**

The IS does not experience additive ROM or stress in the intervertebral disc or facet joints after skip-level arthroplasty, which has fewer biomechanical effects on the IS than does skip-level fusion. This study provides a biomechanical rationale for arthroplasty in treating patients with skip-level cervical degenerative disc disease.

## Background

Anterior cervical discectomy and fusion (ACDF) is the accepted surgical procedure for cervical degenerative disc disease (CDDD) and spondylosis. Following ACDF, motility at the operated level decreases while compensatory motion and intradiscal pressure (IDP) at the adjacent level increase, which are important factors in accelerating adjacent segment degeneration (ASD) [1]. Several biomechanical studies have demonstrated that a multilevel fused mass has greater effects on the adjacent levels than a one-segment fusion does [2–5]. Cervical disc arthroplasty (CDA), as an alternative procedure to ACDF, has gained increased popularity worldwide since the first artificial device was approved by the U.S. Food and Drug Administration (FDA) in 2007 [6, 7]. Most previous studies have investigated the biomechanical effects of single-level or contiguous two-level CDA and suggested that the postoperative kinematics and IDP of the adjacent levels were similar to those of the normal cervical spine due to the motion-preserving property of the procedure at the operated level [5, 8–11].

Skip-level CDDD is a unique type of multilevel CDDD that results in degenerative conditions in two nonadjacent segments. Increased attention has focused on an optimal surgical strategy for patients with skip-level CDDD. Although three-level anterior arthrodesis may avoid extra forces on the intermediate segment (IS) from fused masses on two sides, this fusion construct is associated with a decreased fusion rate and sacrifices the normal IS [12, 13], making three-level anterior arthrodesis a suboptimal treatment option. Noncontiguous ACDF with two cervical plates or anchored cages is an alternative option for the treatment of skip-level CDDD patients. However, the accelerated rate of IS degeneration ranges from 6.25 to 20% [14, 15]. Due to its motion-preserving properties, CDA may theoretically be the best surgical procedure to treat skip-level CDDD. However, evidence is minimal in terms of skip-level CDA, and the biomechanical effects of CDA on the IS are currently unclear.

Although biomechanical alteration after multilevel fusion has been studied, most studies have examined contiguous cervical levels. Finn et al. [16] performed a cadaveric study to determine biomechanical changes in the IS after skip-level fusion and demonstrated that the range of motion (ROM) of the IS increased by 35%. However, this study lacked quantitative analysis of the stress on the IS and facet joints. Qualitatively, the biomechanical response of the IS following CDA is expected to be similar to that of the intact cervical spine. To the best of our knowledge, limited biomechanical data are available that examine the effects of CDA on the IS of the cervical spine. The primary objective of this study was to investigate the biomechanical and kinematic changes resulting from skip-level fusion and skip-level arthroplasty constructs, especially on the IS, in the cervical spine. This study attempts to fill this knowledge gap.

## Methods

A geometrically three-dimensional finite element (FE) model of the cervical spine (C2-C7) was constructed and validated in our previous study [17]. The mid-sagittal symmetrical model was constructed on the basis of a 28-year-old healthy male volunteer (165 cm, 65 kg) without cervical disease [17]. CT scans with a 0.75-mm thickness and a 0.69-mm interval were obtained using a CT scanner (SOMATOM Definition AS+, Siemens, Germany).

### Construction of the cervical spine model and instruments

The CT images were imported into Mimics 17.0 (Materialize Inc., Leuven, Belgium) to reconstruct the geometric structure of the C2-C7 cervical vertebrae and output STL files. Next, the reconstructed model was embedded into Geomagic Studio 12.0 (3D System Corporation, Rock Hill, South Carolina, USA) to create a symmetrical model. This model was processed using CATIA v5r21 (Dassault systems Corporation, Velizy-Villacoublay Cedex, France) to optimize the structure by denoising, surfacing and smoothing. Then, the model was imported into Hypermesh 12.0 (Altair, Troy, MI, USA) to develop a high-quality FE mesh. Finally, the C2-C7 model was imported into ABAQUS 6.9.1 (Dassault Systems Corporation) to set boundary conditions and perform the analysis.

In this FE model, a 0.4-mm-thick shell composed of cortical bone and vertebral endplates was constructed [17, 18]. The intervertebral disc was partitioned into the annulus fibrosus and nucleus pulposus at a volume ratio of 6:4 [17, 18]. Annulus fibers, which are embedded in a matrix of annulus ground substances and account for approximately 19% of the entire annulus fibrosus volume, were constructed with an inclination to the transverse plane between 15° and 30° [18]. The gap of the facet joint was 0.5 mm, and each articular process was covered by an articular cartilage layer with nonlinear surface-to-surface contact [17]. Additionally, five groups of ligaments including the anterior longitudinal ligament (ALL), posterior longitudinal ligament (PLL), ligamentum flavum, interspinous ligament and capsular ligament were attached to the corresponding vertebrae using tension-only truss elements.

Two well-known devices, the Zero-P system (Synthes, Oberdorf, Switzerland) and the Prestige-LP System (Medtronic Sofamor Danek, Memphis, Tennessee) were employed in this study. The dimensions (width, length and height) of the Prestige-LP and cage were 15 mm, 16 mm and 6 mm, respectively. The self-tapping screws were 6.5-mm long (recommended by manufacturers of such stabilization cages). The FE models of the devices were constructed using a computer-aided design (CAD). The material properties and mesh types are listed in Table 1 [17–20]. The number of elements and nodes of the cervical spine model are presented in Table 2.

**Table 1:** Material properties and mesh types of the cervical finite element model

	Yong modulus (MPa)	Poisson ration	Element type	Cross section (mm^2^)
Cortical bone	12,000	0.29	C3D4	–
Cancellous bone	200	0.29	C3D4	–
Annulus fibrosus substance	4.2	0.49	C3D4	–
Annulus fibers	450	0.45	T3D2	–
Nucleus pulposus	1	0.45	C3D4	–
Facet joint cartilage	10.4	0.4	C3D4	–
ALL	30	0.3	T3D2	6.1
PLL	20	0.3	T3D2	5.4
LF	1.5	0.3	T3D2	50.1
IL	1.5	0.3	T3D2	13.1
SL	1.5	0.3	T3D2	13.1
CL	10	0.3	T3D2	46.6
Titanium	110,000	0.3	C3D4	–
PEEK	3600	0.3	C3D4	–

ALL, anterior longitudinal ligament; CL, capsular ligament; IL, interspinous ligament; LF, ligamentum flavum; PLL, posterior longitudinal ligament; SL, supraspinous ligament; C3D4, tetrahedron; T3D2, truss, tension only

**Table 2:** The number elements of and nodes for cervical spine model

	Element	Node
C2	94,104	166,051
C3	65,712	117,362
C4	77,714	138,536
C5	75,166	133,827
C6	83,609	147,488
C7	101,499	178,994
C2/3	8364	15,502
C3/4	6122	11,425
C4/5	8217	15,930
C5/6	4649	8894
C6/7	9514	17,512
ALL	125	126
PLL	117	118
LF	103	104
IL	65	66
SL	35	36
CL	201	202

ALL, anterior longitudinal ligament; CL, capsular ligament; IL, interspinous ligament; LF, ligamentum flavum; PLL, posterior longitudinal ligament; SL, supraspinous ligament

### Boundary conditions

The boundary conditions correspond most commonly to constraint literature. A tie connection was defined between intervertebral discs and adjacent endplates. Frictionless contact was applied between the articular surfaces of the facet joints. The cancellous bone graft filled the combined cage. A nonbonded contact was applied between the supra- and infra-surfaces of the cage and the relevant vertebral surfaces with a contact friction coefficient of 0.3 [21]. A tie constraint was applied to graft-vertebrae and screw-vertebrae interfaces to simulate rigid fusion. The implant vertebrae in the arthroplasty model were applied with tie constraints to simulate full osseointegration [22]. The implant-implant interfaces of the artificial cervical disc were designated with surface-to-surface sliding contact with a fraction coefficient of 0.07 [23].

### Experimental conditions

**Fig. 1:**
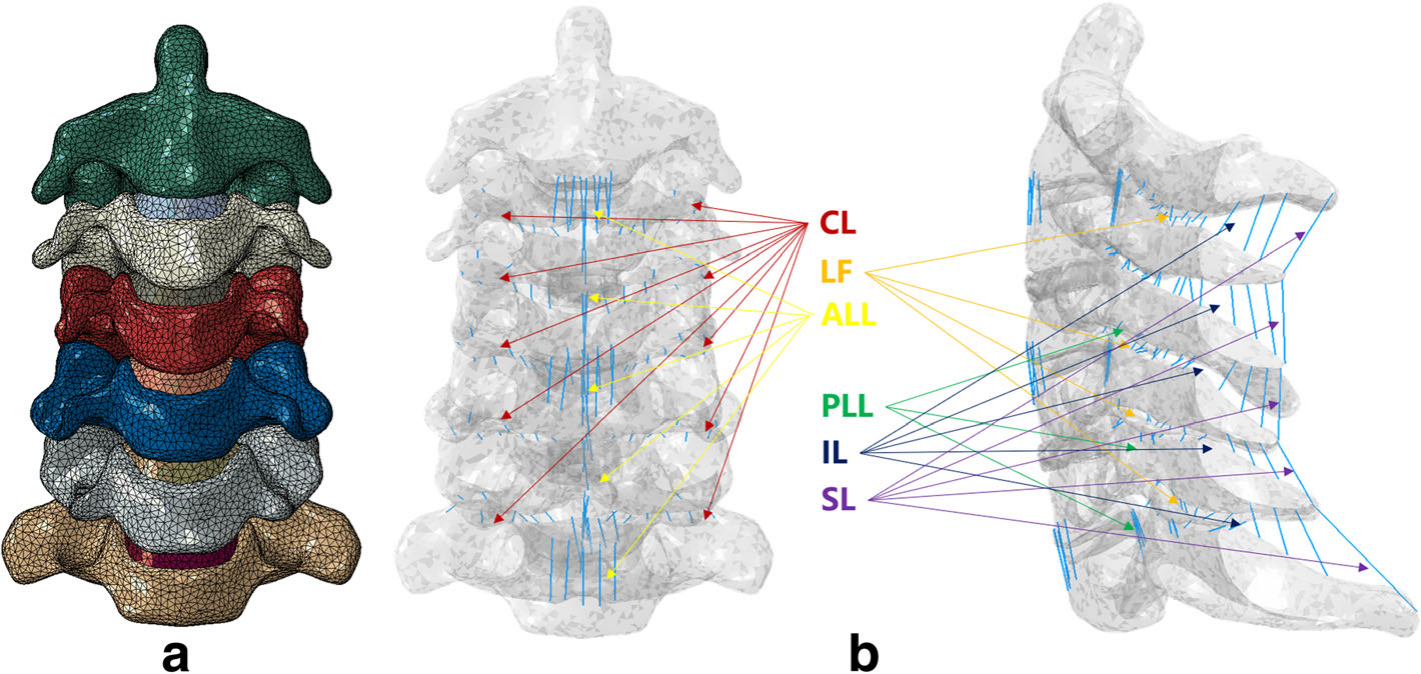
Finite element of a healthy cervical spine (C2-C7). ALL, anterior longitudinal ligament; CL, capsular ligament; IL, interspinous ligament; LF, ligamentum flavum; PLL, posterior longitudinal ligament; SL, supraspinous ligament

The intact cervical spine was fixed at the inferior endplate of C7 with six degrees of freedom. Follower loads of 75 N were used to simulate muscle force and head weight. A 1.0-N·m moment was applied on the top of the C2 vertebral to produce flexion, extension, lateral bending and axial rotation (Fig. 1). The ROM of each segment was calculated and validated by previously published data.

Based on our previous study, we chose C3/4 and C5/6 as the implanted levels because they are the most frequently involved levels in clinical practice [24]. To reflect the actual surgical procedures in the numerical models, the ALL, PLL, nucleus pulposus, and annulus fibrosus were resected at C3/4 and C5/6 while the lateral structures such as the uncinate processes were preserved. In the fusion model, Zero-P devices were inserted at the corresponding locations. To simplify the model, shared nodes at the screw-plate interfaces were used, thus not allowing relative motion between the components. A fully bonded contact between the screw surface and vertebral cortical shell was created [21]. For the arthroplasty model, the Prestige-LP disc was inserted at the corresponding location.

**Fig. 2:**
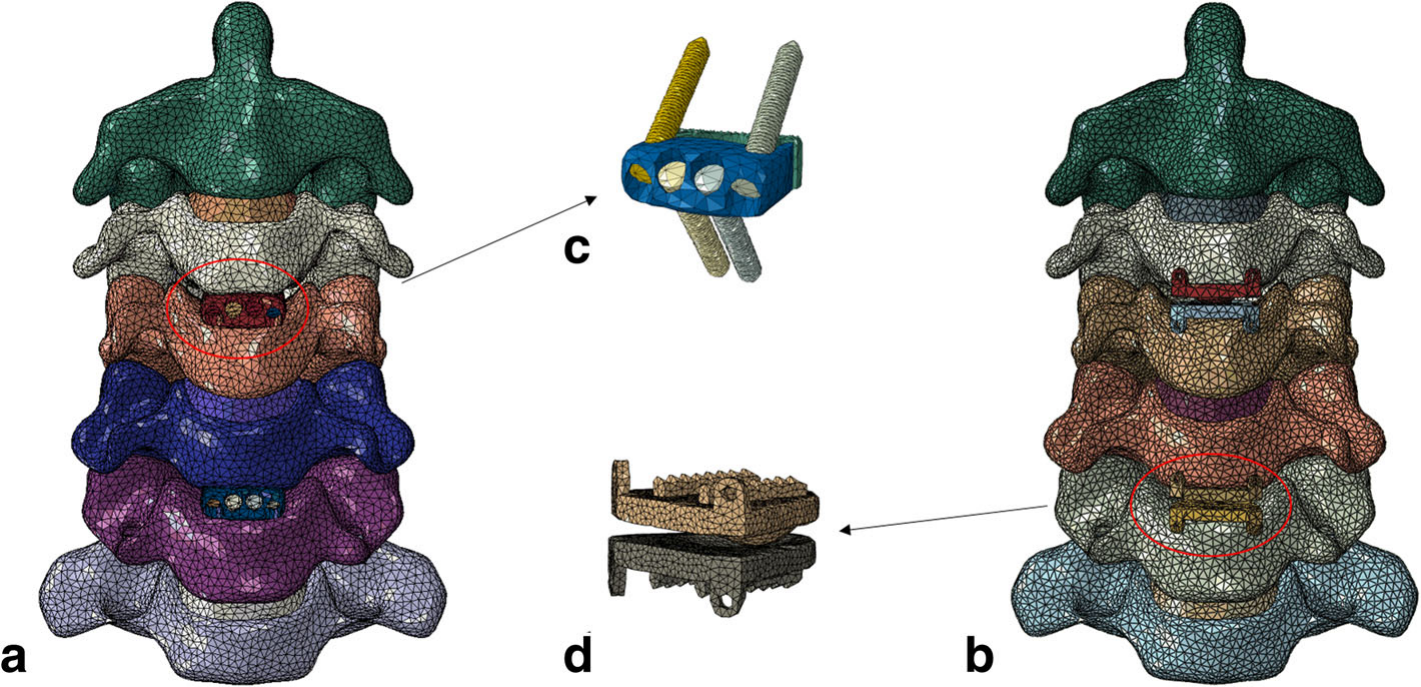
Two surgical models were performed at C3/4 and C5/6. (**a**) Skip-level fusion with the Zero-P device, (**b**) skip-level arthroplasty with the Prestige-LP device, (**c**) Zero-P device and (**d**) Prestige-LP device

The displacement-control test protocol was conducted in subsequent reconstructions. The end-point in each direction corresponding to the intact cervical model formed the basis for the subsequent displacement-control testing conditions including (1) skip-level fusion at C3/4 and C5/6 and (2) skip-level arthroplasty at C3/4 and C5/6 (Fig. 2). This method is based on the assumption that a patient will attempt to move the cervical spine in a manner similar to their preoperative motion capabilities.

## Results

### Validation of the intact cervical model

**Fig. 3:**
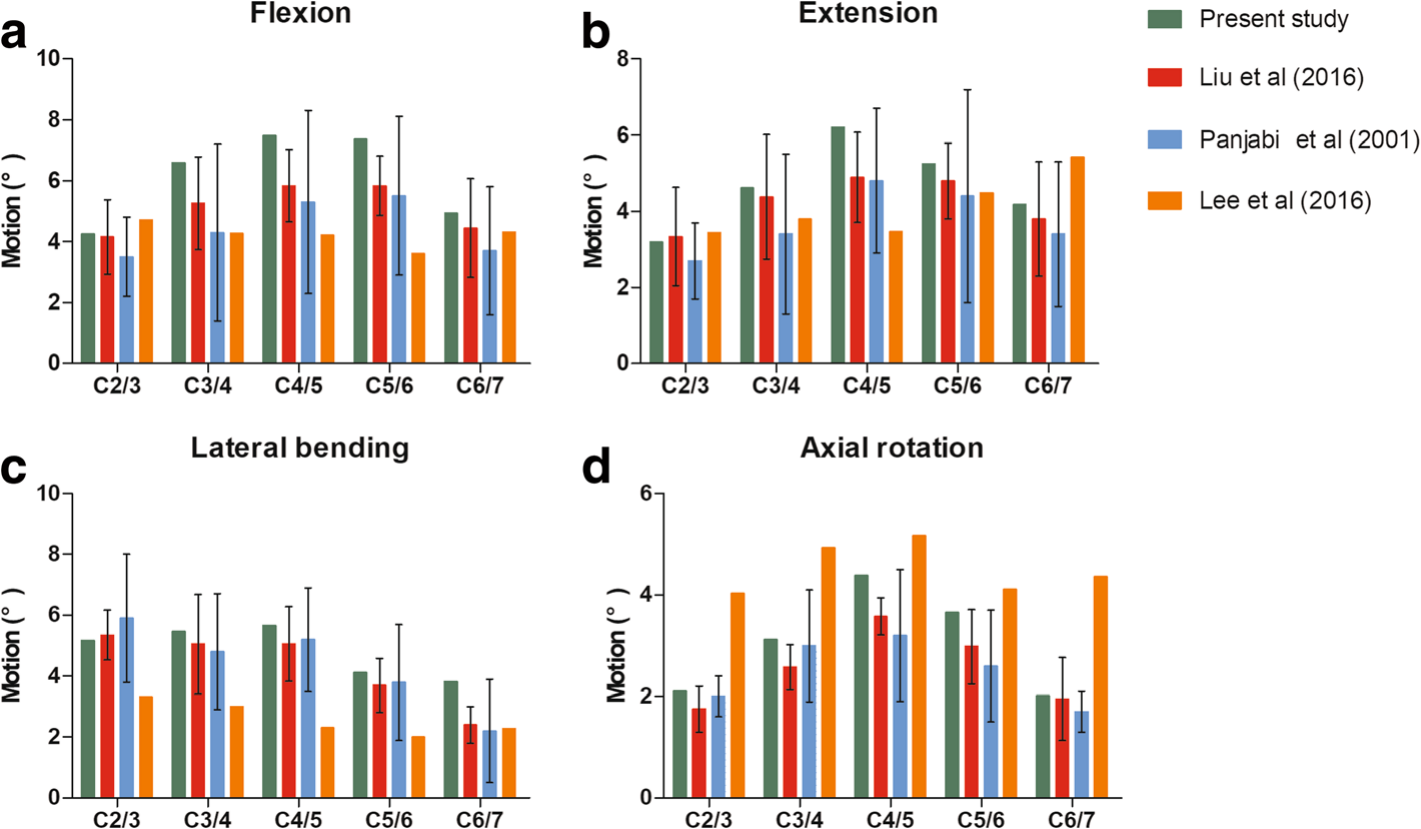
The predicted ranges of motion (ROMs) are validated by previous published data

The ROM of each segment in the intact cervical spine model subjected to a 1.0-N·m moment is shown in Fig. 3. The predicted ROMs for all levels were compared with those in previous biomechanical and FE analysis studies [25–27]. The ROMs of the intact model at C2/3, C3/4, C4/5, C5/6 and C6/7 were 4.25°, 6.59°, 7.47°, 7.36° and 4.94°, respectively, in flexion; 3.19°, 4.62°, 6.21°, 5.25° and 4.18°, respectively, in extension; 5.17°, 5.46°, 5.66°, 4.12° and 3.83°, respectively, in lateral bending; and 2.11°, 3.12°, 4.39°, 3.65° and 2.02°, respectively, in axial rotation. The ROMs in the current study were similar to those in previous studies.

### Range of motion at the operated levels

**Fig. 4:**
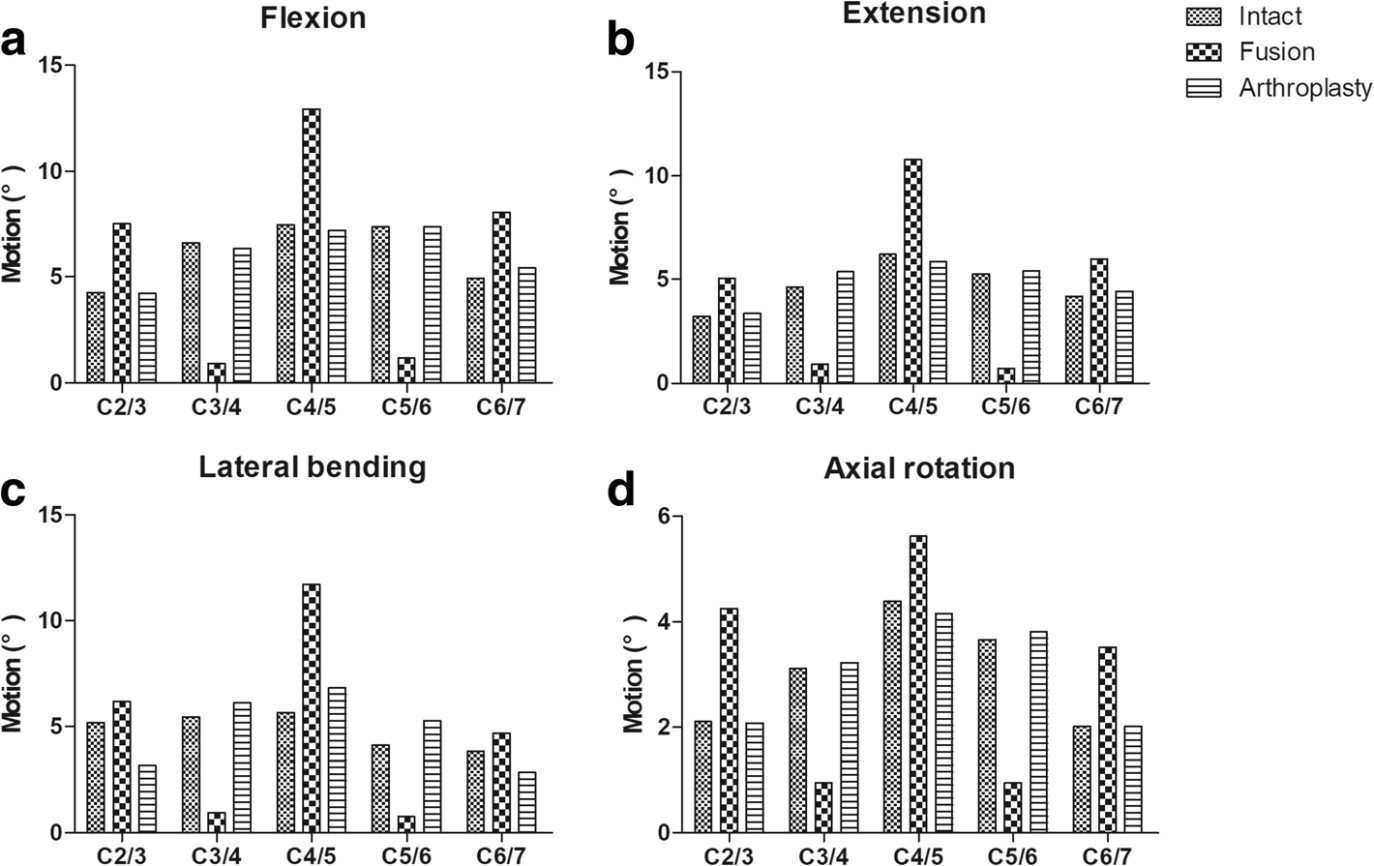
The segmental range of motion (ROM) under different moments. (**a**) Flexion, (**b**) extension, (**c**) lateral bending and (**d**) axial rotation

In the fusion model, the ROMs of the fusion levels (C3/4 and C5/6) were markedly decreased in all directions. The ROMs of the arthroplasty model were similar to those of the intact spine. At the C3/4 level, the ROMs increased by 3.9, 16.2, 12.1 and 3.2% in the flexion, extension, lateral bending and axial rotation directions, respectively, compared to those in the intact cervical spine model. At the C5/6 level, the ROMs increased by 6.9 and 12.8%, 27.7 and 4.6% in flexion, extension, lateral bending and axial rotation, respectively, compared to the ROMs in the intact cervical spine (Fig. 4).

### Range of motion at the adjacent and intermediate levels

In the fusion model, compared to the intact cervical spine model, the increases in the ROMs of the superior adjacent level (C2/3) ranged from 29.0 to 77.7%; the maximum increase was observed for axial rotation. Increases in the ROMs of the intermediate level (C4/5) ranged from 39.4 to 89.2%; the maximum increase was observed for lateral bending. Increases in the ROMs of the inferior adjacent level (C6/7) ranged from 35.2 to 74.3%; and the maximum increase was observed in axial rotation.

In the arthroplasty model, the ROM of the superior adjacent level (C2/3) increased by 0.5% in extension and decreased by 0.5, 28.8 and 1.4% in flexion, lateral bending and axial rotation direction, respectively. The ROM of the intermediated level (C4/5) increased by 0.3% in lateral bending and decreased by 3.5, 5.5 and 5.2% in flexion, extension and axial rotation, respectively. The ROM of the inferior adjacent level (C6/7) decreased by 9.9, 5.9, 12.5 and 0.5% in flexion, extension, lateral bending and axial rotation, respectively (Fig. 4).

### Intradiscal pressure in the adjacent and intermediate levels

**Fig. 5:**
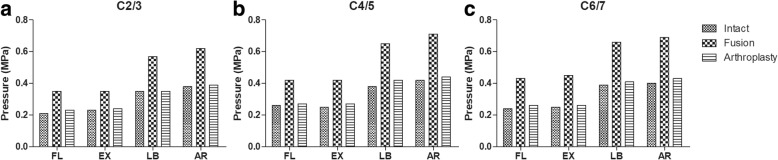
The maximum intradiscal pressure (IDP) at untreated levels. (**a**) C2/3, (**b**) C4/5 and (**c**) C6/7. FL Flexion, EX extension, LB lateral bending, AR axial rotation

In the intact model, the IDPs at C2/3 were 0.21 MPa, 0.23 MPa, 0.35 MPa and 0.38 MPa under flexion, extension, lateral bending and axial rotation moments, respectively. The corresponding IDPs at C4/5 were 0.26 MPa, 0.25 MPa, 0.38 MPa and 0.42 MPa, respectively. The corresponding IDPs at C6/7 were 0.24 MPa, 0.25 MPa, 0.39 MPa, and 0.40 MPa, respectively. In the fusion model, the IDPs at C2/3 increased by 66.7, 52.2, 62.9 and 63.2% under flexion, extension, lateral bending and axial rotation moments, respectively, compared to the IDPs in the intact model. The corresponding IDPs at C4/5 increased by 61.5, 68.0, 71 and 69%, respectively. The corresponding IDPs at C6/7 increased by 79.2, 80.0, 69.2 and 72.5%, respectively. In the arthroplasty model, the IDPs at C2/3 increased by 9.5, 4.3, 5.4 and 2.6%, under flexion, extension, lateral bending, and axial rotation moments, respectively, compared to the IDPs in the intact model. The corresponding IDPs at C4/5 increased by 3.8, 8.0, 10.5 and 4.7%, respectively. The corresponding IDPs at C6/7 increased by 8.3, 4.0, 5.1 and 7.5%, respectively (Fig. 5).

### Facet contact force at the adjacent and intermediate levels

**Fig. 6:**
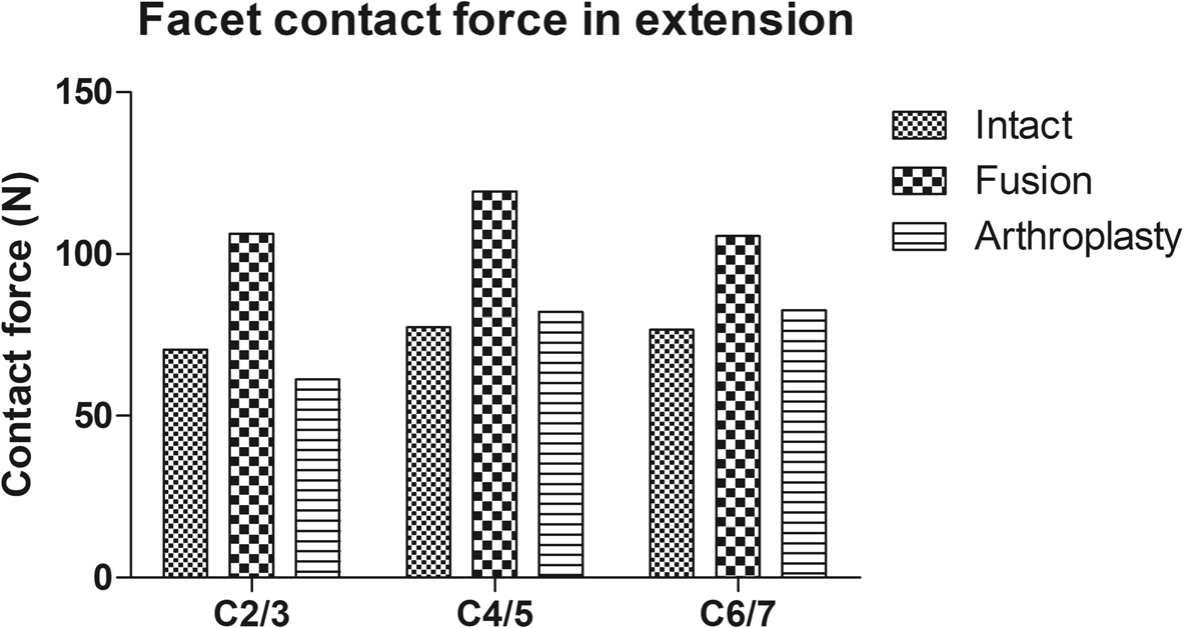
The maximum facet contact force (FCF) at unoperated levels in extension

The maximum facet contact force at C2/3, C4/5 and C6/7 was noted at the end of extension. In the intact model, the facet contact forces at C2/3, C4/5 and C6/7 under extension were 70.57 N, 77.39 N and 76.71 N, respectively. In the fusion model, the maximum facet contact forces increased by 36.5, 54.2 and 37.4% at C2/3, C4/5 and C6/7, respectively, compared to those in the intact model. In the arthroplasty model, the maximum facet contact force decreased by 13.0% at C2/3 and increased by 6.4 and 7.6% at C4/5 and C6/7, respectively, compared to those in the intact model (Fig. 6).

## Discussion

Whether ASD is a consequence of the natural degenerative process or a result of cervical fusion is still under debate [28, 29]. However, abnormal IDP at levels adjacent to a fusion may be an important factor for the development of degenerative changes. Avascular nutrition exchange within the disc space is dependent primarily on diffusion and osmotic gradients. Sustained increases in mechanical loading have been shown to impair the diffusion of nutrients entering the disc, resulting in accelerated disc degeneration [30–32]. Moreover, a previous study indicated that progressive cervical disc degeneration occurs most frequently at the level immediately adjacent to a fusion [28]. This observation may imply that segments immediately adjacent to fusion may reflect a predisposition for progressive degeneration; therefore, degeneration of the intermediated disc, which is immediately adjacent to two fusion masses, may be accelerated in skip-level fusion compared to that in other levels. Our results showed that the ROM of flexion/extension was almost uniformly changed, whereas the ROMs of lateral bending and axial rotation were unevenly affected over the remaining unfused levels in the fusion model. The ROM of the IS in lateral bending was increased by up to 4–5 times that of the inferior and superior segments. This result was consistent with that in a previous study by Finn et al. [16], which indicated that biomechanical effects were different following a fusion construct at different moments. Moreover, in the current study, the IDPs of the unfused levels markedly increased and were evenly distributed across the remaining mobile segments, while the facet contact force of the IS increased by 1.5 times that of the inferior and superior segments. These results reveal that although increased biomechanical forces following skip-level fusion were not additive on discs when two fusions were performed on either side of an IS, the stress on the intermediate-level facet joints was markedly increased. Facet degeneration has been shown to be a main cause of neck pain. Abnormal loading has been associated with the development of facet degeneration [33, 34]. Thus, it is reasonable to speculate that the degenerative process may be accelerated in facet joints following skip-level fusion. However, further long-term clinical observations are needed to verify this hypothesis.

Theoretically, CDA maintains motion at a decompressed interspace, resulting in improved load transfer and reduced stress on the adjacent intervertebral discs. Although previous cadaveric biomechanical studies and FE studies have demonstrated superior kinematics and biomechanical effects after a CDA construct, most studies investigated one-level or contiguous two-level CDA [5, 10, 11, 35]. Because the mechanism of skip-level arthroplasty is different from that of contiguous two-level CDA, conclusions related to the biomechanical effects based on contiguous two-level CDA may not be applicable to skip-level CDA. The present study revealed a slight increase in the segmental ROMs in all directions at the implanted levels and a reduction in motion at the untreated levels in the arthroplasty model. This result may reflect the restoration of motion after CDA, and the findings are consistent with those of previously published studies that used an FE model of the cervical spine following one- or two-level CDA [25, 36]. This phenomenon may result from resection of the supporting structures such as the ALL and PLL. Clinically, our previous results revealed that after skip-level CDA, the ROM showed a slight increase at the operated levels [24]. Contiguous two-level CDA follow-up results also showed a slight increase in the ROMs at the operated levels over a longer follow-up period [37, 38]. Accordingly, the present data match well with the results observed under in vivo conditions. In 2005, Dmitriev et al. [5] first reported that CDA preserved adjacent segment IDP and maintained kinematics near preoperative values at treated and adjacent segments. Pimenta et al. [39] documented that each treated level following CDA was biomechanically distinct, independent of the adjacent level. Therefore, there is less potential transmission of load and stress to the adjacent levels following CDA. In the present investigation, the IDPs were slightly increased in the superior, intermediate and inferior adjacent segments and evenly distributed across these levels. Compared to the intact model, the facet contact forces were slightly increased at C4/5 and C6/7 and decreased at C2/3. Furthermore, the IDP and facet contact force at the IS in the fusion model were 1.5 times those of the arthroplasty model. These findings indicate that CDA had less impact on the biomechanical environment of the adjacent segments than fusion did and CDA may protect the IS from the development of disc degeneration.

In the current study, the authors used a hybrid loading condition that was first proposed by Panjabi [40] to simulate motion redistribution in a biomechanical test, and this is the best tool available to evaluate adjacent level effects. The rationale for this method is that the adaptive response of the spinal system attempts to restore the spine to its natural state [40]. Further studies by Lee et al. [41] reported that specimens under a displacement-control condition may represent the condition of a cervical spine after a longer follow-up period. Actual in vivo loading at adjacent untreated levels after surgery is likely to be a hybrid loading type, as patients adapt over time to their postoperative conditions. In recent decades, several studies have successfully used the hybrid method. Gandhi et al. [10] conducted a cadaveric study to evaluate biomechanical trends at treated and adjacent levels after single-level, two-level and hybrid constructs were employed at C2-T1. Faizan et al. [36] used a validated C3-C7 FE model to evaluate adjacent-level effects after two-level fusion, two-level arthroplasty and hybrid constructs.

This study has several limitations. Although FE analysis is a useful computational modeling tool, the results should be interpreted with caution. First, it is important to note that these results were based on a single FE model used to simulate skip-level fusion and arthroplasty constructs. The biomechanics of this model may not be analogous to the pathology of the cervical spine in vivo when two- or more ISs are bordered by two operated levels or when the IS located at a level of the cervical spine other than C4/5. This study aims to provide a direction rather than actual data and other situations should be considered in future studies. Second, simplified parameters such as material properties, boundary conditions, frictionless contact, musculoskeletal systems and implanted locations create idealized conditions that lead to perfect outcomes and cannot completely represent actual in vivo conditions after surgery. The bone-implant interface is much more complicated, and the graft bone and cage/screws are thoroughly fused. The present results may provide clinically relevant insights into cervical spine biomechanics. Third, the surgical insertion procedure is simplified in the models, in which the distraction of vertebrae before inserting a device was not involved. This may not be a realistic assumption.

## Conclusion

The FE results indicate that skip-level arthroplasty produces biomechanical and kinematic properties similar to those of the intact spine. The biomechanics and kinematics are markedly altered in the skip-level fusion construct. In the arthroplasty model, the IS does not exhibit an additive ROM or additive stress in the intervertebral disc or facet joints compared to those in the superior and inferior adjacent segments. However, in the fusion model, the ROMs were unevenly redistributed across the unfused levels, and the IS experienced additive stress at the facet joints. Overall, based on the results of this FE study, skip-level arthroplasty has a reduced biomechanical impact on the IS compared to skip-level fusion. This study provides a biomechanical rationale for the use of CDA to treat patients with skip-level CDDD.

## Abbreviations

ACDF: Anterior cervical discectomy and fusion
ALL: Anterior longitudinal ligament
ASD: Adjacent segment disease
CAD: Computer-aided design
CDA: Cervical disc arthroplasty
CDDD: Cervical degenerative disc disease
CT: Computed tomography
FE: Finite element
IDP: Intradiscal pressure
IS: Intermediate segment
PLL: Posterior longitudinal ligament
ROM: Range of motion
